# Rapid Two-Temperature Formalin Fixation

**DOI:** 10.1371/journal.pone.0054138

**Published:** 2013-01-18

**Authors:** David Chafin, Abbey Theiss, Esteban Roberts, Grace Borlee, Michael Otter, Geoffrey S. Baird

**Affiliations:** 1 Ventana Medical Systems, Inc., Tucson, Arizona, United States of America; 2 Department of Laboratory Medicine, University of Washington, Seattle, Washington, United States of America; 3 Department of Pathology, University of Washington, Seattle, Washington, United States of America; Moffitt Cancer Center, United States of America

## Abstract

Formalin fixation is a mainstay of modern histopathologic analysis, yet the practice is poorly standardized and a significant potential source of preanalytical errors. Concerns of workflow and turnaround time drive interest in developing shorter fixation protocols, but rapid protocols can lead to poor histomorphology or inadequate downstream assay results. Additionally, assays such as immunohistochemistry for phosphorylated epitopes have historically been challenging in the context of formalin-fixed tissue, indicating that there may be room for improvement in this process that is fundamental to the practice of anatomic pathology. With these issues in mind, we studied basic formalin biochemistry to develop a novel formalin fixation protocol that involves a pre-incubation in subambient temperature formalin prior to a brief exposure to heated formalin. This new protocol is more rapid than standard protocols yet preserves histomorphology and yields tissue that is compatible with an expanded set of downstream clinical and research assays, including immunohistochemistry for phosphorylated epitopes.

## Introduction

In the 1890s, the industrial precursor to Schering AG patented an aqueous solution of gaseous formaldehyde with the name “formalin,” and for more than a century, histologists have used formalin to prepare tissue for sectioning and microscopic examination [1]. Despite this long history of use, the chemistry of formaldehyde’s interactions with tissue constituents is understood less completely than one might expect. What has been thought to occur when formalin fixes tissue is that formaldehyde crosslinks proteins by creating methylene bridges between adjacent amino groups on proteins, thereby lending the tissue structural integrity as well as inactivating potentially destructive enzymes within the tissue. This is an oversimplification, however, and the interactions of formaldehyde with every type of biomolecule in a tissue are still poorly understood. Tissue proteins, for example, are traditionally thought to be simply cross-linked by formaldehyde, but they can participate in more chemical reactions with formaldehyde than just cross-linking. Protein amino groups are not the sole targets of formalin as evidenced by the observations that peptides lacking amino groups can be reversibly “fixed” [2] and non-peptides such as nucleic acids also appear to be altered by fixation [3] by incompletely understood mechanisms.

The behavior of formaldehyde in tissues is further muddied by the fact that formaldehyde in solution is, in fact, mostly not formaldehyde. Rather, it is in equilibrium with a large excess of its nonreactive hydrate [4], methylene glycol, such that only a tiny fraction of the molecules in “formalin” are reactive formaldehyde species. This latter fact may account for the oft-repeated observation that formalin penetrates tissues briskly but fixes them slowly [3]. Despite these mechanistic uncertainties, formalin remains an overwhelmingly popular choice of fixatives in clinical laboratories [5], despite the existence of multiple alternative fixation strategies [6]–[11].

The search for ever-faster formalin fixation protocols has been driven historically by considerations of overall laboratory turnaround time and workflow, as formalin fixation and tissue processing can comprise the majority of the time required for histopathologic analysis. Commercial tissue processors designed to address this need fix tissue rapidly by applying formalin and external energy in the form of heat or microwaves [12]–[24]. Heat likely speeds fixation for two reasons: first, the formaldehyde-methylene glycol equilibrium shifts towards formaldehyde at higher temperatures and raises the effective concentration of the active molecule [4], and second, protein crosslinking, like many other chemical reactions, should proceed more quickly at elevated temperatures. Ultrasound has been applied to fixation, as well, as an alternate mechanism to impart energy to speed fixation [25]–[29].

While accelerated fixation with heated formalin has potential benefits in decreasing clinical turnaround times, our experience and that of others indicates that the quality of histologic and molecular studies suffers in heated protocols [1], [30]–[32]. Additionally, it is concerning that outside of a few clinical applications, tissue fixation is not rigidly standardized in the clinical laboratory. One laboratory may choose to fix clinical tissue samples rapidly at an elevated temperature and another may choose to fix slowly at ambient or low temperatures, but both preanalytical conditions are expected to perform adequately for downstream tissue-based assays. The influence of variation in preanalytical specimen preparation is increasingly being recognized as a major problem in diagnostic pathology [33]–[35], as it prevents accurate targeted analysis of a patient’s specimen and elucidation of the best possible therapy for a disease, both essentials in the paradigm of personalized medicine. Posttranslational protein modifications such as phosphorylation, for example, are known to be important indicators of signaling pathway activity and represent promising clinical biomarkers, but they can be very difficult to measure in fixed tissues because of preanalytical variables [36]–[40]. Formalin fixation prior to phosphoprotein analysis must crosslink tissue proteins and inactivate the phosphatase enzymes that might remove protein phosphates; failure on either of these tasks in inappropriately fixed tissue makes it impossible to interrogate these pathways and use such assays as diagnostics.

Standardized fixation parameters have been adopted for some clinical biomarker assays to mitigate the errors stemming from preanalytical variation, such as the American Society of Clinical Oncology/College of American Pathologists (ASCO/CAP) guidelines for HER2 IHC that call for fixation in neutral buffered formalin for at least 6 hours and no more than 48 hours. While these guidelines are well intentioned, they still allow an 8-fold variation in fixation time and are not meant to represent optimal conditions for all IHC assays. It is highly unlikely, however, that the large number of different laboratory-developed IHC assays performed clinically around the world could be validated for all potential fixation protocols. Faced with this problem, we sought to identify a standardized formalin fixation protocol that was applicable to a broad range of tissue types and optimized both for speed and preservation of diagnostic molecular features. Beginning with basic protein biochemistry studies, we rationally developed a novel fixation protocol based on formalin biochemistry, demonstrated its broad applicability to standard histopathology, and finally showed that the novel protocol provided acceptable IHC assay results for numerous targets including several phosphoprotein assays.

## Materials and Methods

### Tissue Samples

De-identified unfixed human tonsil tissue samples were obtained from either the Cooperative Human Tissue Network (CHTN, Nashville, TN) or Bio-Options Inc. (Brea, California); surgical samples were wrapped in saline-soaked gauze and kept on wet ice overnight prior to use. Human colon carcinoma samples for phosphoprotein analysis were obtained from Indivumed GmBH (Hamburg, Germany) as paraffin-embedded samples; samples from Indivumed were used because the cold ischemia time offered by this tissue vendor were superior (shorter) than possible from other vendors or in our clinical area. Indivumed scientists performed the fixation protocols described on these colon carcinoma samples. Calu-3 xenograft tumors [41] were provided by the Assay Development Support Service (ADSS) at Ventana Medical Systems, Inc. (Tucson, AZ). Additional tissue samples were collected from surgeries at the author’s (GSB) institution under a waiver of consent, using procedures approved by the University of Washington Institutional Review Board. Upon excision, fresh tissue was carried to the pathology laboratory, generally within 30–60 minutes, and after the diagnostic pathologist had taken sections needed for diagnosis, excess tissue was dissected into several equivalently sized portions for each fixation condition. Tissue specimens were limited to 1 cm in maximum dimension, and never exceeded 4 mm in thickness. For comparison of histomorphology and IHC between tissue fixed with experimental conditions and tissue generated by pathology department histotechnologists in a CLIA- and College of American Pathologists (CAP)-certified laboratory, unstained slides from the clinical tissue block generated in each case (10–48 hours RT formalin fixation) were collected.

### Fixation Protocols

For all experiments, fixation consisted of immersion in 10% neutral buffered formalin (Saturated aqueous formaldehyde from Fisher Scientific, Houston, TX, buffered to pH 6.8–7.2 with 100 mM phosphate buffer) for 0–24 hours at temperatures ranging from 4**°**C to 50**°**C. Room temperature ranged from 20–25**°**C. Tissue samples collected from clinical cases were fixed at room temperature for 0, 2, 4 or 24 hours, or else 2 hours in 4**°**C formalin followed by 2 hours in 45**°**C formalin, the latter protocol being termed “2+2”. The 0 hour formalin specimen was placed directly into 70% ethanol. Fixation was carried out in 100–500 mL covered beakers in a refrigerator for 4**°**C treatment, in a fume hood for room temperature treatment, and in a fume hood on a hot plate for elevated temperatures (i.e. 45**°**C). 2+2 samples were carried from the refrigerated beaker to the fume hood for sequential temperature treatments. Clinical blocks were fixed at room temperature for 10–48 hours in the clinical laboratory, with 10 hours being the minimum for weekday samples and 48 hours only used for samples fixed over weekends. To facilitate processing all research samples from a day on the same processor cycle, all samples were stored in 70% ethanol after variable-length fixation prior to starting the tissue processor (Peloris, Leica, Germany). The processor run was programed as follows: 80% ethanol for 30 minutes, 95% ethanol for 60 minutes, 100% ethanol for 3 hours, xylene for 3 hours, and paraffin for 3 hours 20 minutes. Clinical blocks were processed by the same protocol without a variable 70% ethanol preincubation. All solvent processor steps were kept at 45**°**C under ambient pressure, and the paraffin step was held at 60**°**C under vacuum. All specimens were embedded in paraffin and cut onto glass slides as 5-micron sections for routine Hematoxylin/Eosin (H&E) stain or and 4-micron sections for IHC or molecular studies. The clinical tissue samples collected for this study are enumerated in table 1.

**Table 1:** Anatomic locations and numbers of clinical tissue specimens collected for histologic evaluation.

Anatomic Tissue Source	Individual Patient Samples
skin	4
muscle/tendon	5
stomach (antrum/body)	1
spleen	4
colon	5
uterus (endometrium/myometrium)	3
liver	3
breast/adipose/ductal carcinoma *in situ* (DCIS)	1
nerve	1
small bowel	3
appendix	1
cervix	2
ovary	1
fallopian tube	1
cholangiocarcinoma	1
cervical carcinoma	1
metastatic colon carcinoma	1

### RNase A Crosslinking

5 mg/ml RNaseA (Sigma-Aldrich, St. Louis, MO) was mixed with 10% Neutral Buffered Formalin (Richard-Allan Scientific, Kalamazoo, MI) for 10 minutes. At specified times, samples were diluted 10-fold with ice cold water. A 10 µl aliquot was then removed, mixed with an equal volume of 2× SDS-protein loading buffer, electrophoresed on a 4–15% gradient Tris-HCl-SDS polyacrylamide gel (Bio-Rad, Hercules, CA) and silver-stained according to manufacturer’s directions (EMD, Darmstadt, Germany). Precision Plus molecular weight markers were used as directed (Biorad, Hercules, CA).

### Histologic Assessment

Histologic assessment of fixation quality was scored using a modification of a scoring system published by the manufacturer of the tissue processor used in this study [42], described in table 2. The pathologist grading the histologic appearances was blinded to the fixation protocol for each tissue section. Differences in histologic quality scores were assessed with the Kruskal-Wallis test and Dunn’s post-test to correct for multiple comparisons. For data in Figure S1, subjective histologic quality was assessed by a pathologist and categorized as “unacceptable” (colored red in figure), “acceptable for diagnosis but with recognizable defects in morphology” (colored yellow in figure), and “acceptable” (colored green in figure). To ensure reproducibility in this subjective assessment, samples fixed with different protocols were compared to control samples that had been fixed in room temperature formalin for 0, 2, 4, 8 and 24 hours. The results presented here are representative of 10 repeated experiments for each H&E assessments and 2 repeated experiments for the IHC experiments.

**Table 2:** Criteria used in scoring fixation quality by morphology.

Score	Nuclear Morphology	Cytoplasmic Morphology	Overall Morphology
**2**	Sufficient for Clinical Diagnosis, with noproblems evident	Sufficient for Clinical Diagnosis, with noproblems evident	Sufficient for Clinical Diagnosis, with noproblems evident
**1**	Sufficient for Clinical Diagnosis,but problems are evident	Sufficient for Clinical Diagnosis,but problems are evident	Sufficient for Clinical Diagnosis, butproblems are evident
**0**	Insufficient for Clinical Diagnosis	Insufficient for Clinical Diagnosis	Insufficient for Clinical Diagnosis
**Specific** **Criteria**	Quality/clumping of chromatin, under/overstaining with hematoxylin,vacuolization artifacts	Cell-Cell retraction artifacts, visibility ofintercellular bridges (in epidermis), under/overstaining with Eosin, vacuolizationartifacts	Tissue cracking, uneven staining, absenttissue fragments, wrinkling

Each section was scored 0, 1, or 2 in each of three areas, and the scores were summed to give an overall index of quality.

### Automated Immunohistochemistry Staining

Immunohistochemistry assays were performed on a VENTANA Discovery XT automated staining instrument according to the manufacturer’s instructions. Slides were de-paraffinized using EZprep solution (Ventana Medical Systems, Inc.) at 90**°**C, and all reagents and incubation times were chosen as directed on antibody package inserts. Slides were developed using the OmniMap DAB detection kit (Ventana Medical Systems, Inc.) and counterstained with hematoxylin. Antibodies, clones, and titers are listed in table 3. Antibody titers were determined for each antibody using positive and negative control tissues following the manufacturer’s instructions.

**Table 3:** Antibodies, clones and titers used in this study.

Antibody Target	Clone	Dilution	Phosphorylation Site,if Applicable	Vendor	Cat. #
phospho-AKT	D9E	1∶20	Ser473	Cell Signaling Technologies, Danvers, MA	4060
phospho-PRAS40	C77D7	1∶100	Thr246	Cell Signaling Technologies, Danvers, MA	2997
phospho-MTOR	49F9	1∶200	Ser2448	Cell Signaling Technologies, Danvers, MA	2976
phospho-EIF4G	polyclonal	1∶200	Ser1108	Cell Signaling Technologies, Danvers, MA	2441
phospho-44–42 MAPK	20G11	1∶400	Thr202/Tyr204	Cell Signaling Technologies, Danvers, MA	4376
phospho-S6	D57.2.2E	1∶200	Ser235/236	Cell Signaling Technologies, Danvers, MA	4858
phospho-MSK1	polyclonal	1∶50	Thr581	Cell Signaling Technologies, Danvers, MA	9595
phospho-MEK1/2	166F8	1∶50	Ser221	Cell Signaling Technologies, Danvers, MA	2338
phospho-p38MAPK	12F8	1∶50	Thr180/Tyr182	Cell Signaling Technologies, Danvers, MA	4631
HER-2/neu	4B5	Prediluted		Ventana Medical Systems, Tucson, AZ	790–100
CD31	JC70	Prediluted		Ventana Medical Systems, Tucson, AZ	760–4378
Vimentin	V9	Prediluted		Ventana Medical Systems, Tucson, AZ	790–2917
bcl-2	124	Prediluted		Ventana Medical Systems, Tucson, AZ	790–4464
ER	SP1	Prediluted		Ventana Medical Systems, Tucson, AZ	790–4324

### In situ Hybridization (ISH)

Slides were probed for the HER2 and Chromosome 17 Centromeric region using the INFORM HER2 Dual ISH assay (Ventana Medical Systems, Inc.) on the Discovery XT automated slide stainer according to the manufacturer’s instructions. In these analyses, a black dot corresponds to the HER2 probe and a red dot corresponds to the centromeric region of chromosome 17.

### Ethics Statement

The studies described here that involved human tissue either used de-identified samples from commercial sources [Cooperative Human Tissue Network (CHTN, Nashville, TN), Bio-Options Inc. (Brea, California), and Indivumed GmBH (Hamburg, Germany)] or were performed under procedures approved by the University of Washington’s Institutional Review Board. This study was granted a waiver of consent by the University of Washington Institutional Review Board because it employed only de-identified tissue that would have otherwise been discarded, and thus consent (written or oral) was not obtained from subjects.

## Results

**Figure 1:**
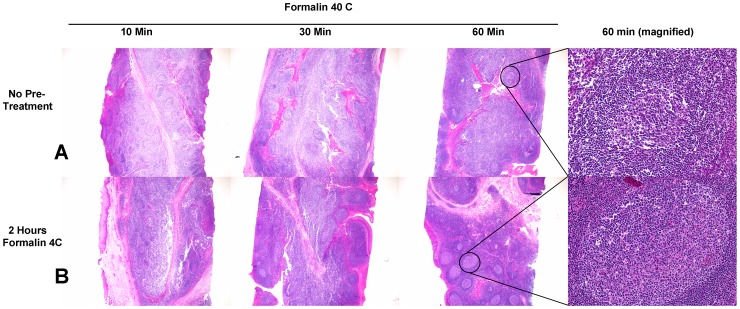
H&E-stained human tonsil samples fixed with heated formalin. A) 4 mm-thick human tonsil samples placed directly into 40°C formalin for 10, 30 or 60 minutes. B) 4 mm-thick human tonsil samples pre-soaked in 4°C formalin for 2 hours and then placed immediately into 40°C formalin for 10, 30 or 60 minutes. Stains are standard H&E. Germinal centers from the center of each 60-minute sample are shown at right in each row, at high magnification, to emphasize morphologic improvements seen with cold preincubation, including amelioration of cytoplasmic retraction artifacts.

**Figure 2:**
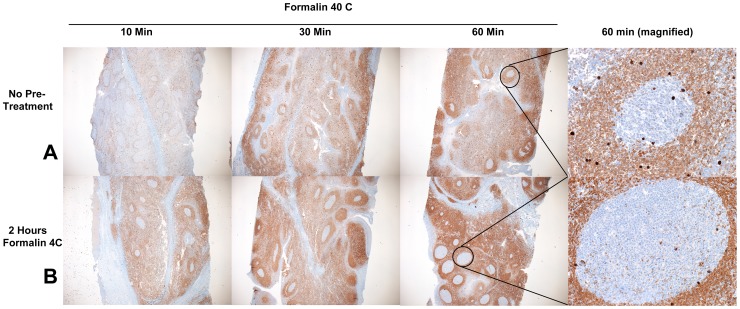
bcl-2 IHC on human tonsil samples fixed with heated formalin. A) 4 mm-thick human tonsil samples placed directly into 40°C formalin for 10, 30 or 60 minutes. B) 4 mm-thick human tonsil samples pre-soaked in 4°C formalin for 2 hours and then placed immediately into 40°C formalin for 10, 30 or 60 minutes. Stains are standard IHC for *bcl-2*. Germinal centers from the center of each 60-minute sample are shown at right in each row, at high magnification, to emphasize darker staining seen with cold preincubation.

One of the problems we noted with rapid heated formalin fixation protocols is that the fixative appears to work very quickly on the outer margins of tissue, but the interior of the tissue appears to be heated immediately and disrupted, before the fixative has had a chance to diffuse throughout the specimen. This results in uneven fixation of tissue, poor morphology and unreliable IHC assay results as seen in row A of both Figures 1 and 2. In this experiment, tissue exposed to 40°C formalin for 60 minutes yielded adequate fixation at the periphery of the tissue but poor histomorphology and immunoreactivity in the center of the tissue. To address this problem, we studied the chemical reactivity of formalin at different temperatures to determine if there was an optimal combination of incubation time and temperature that preserved both histomorphology and immunoreactivity throughout an entire tissue specimen.

**Figure 3:**
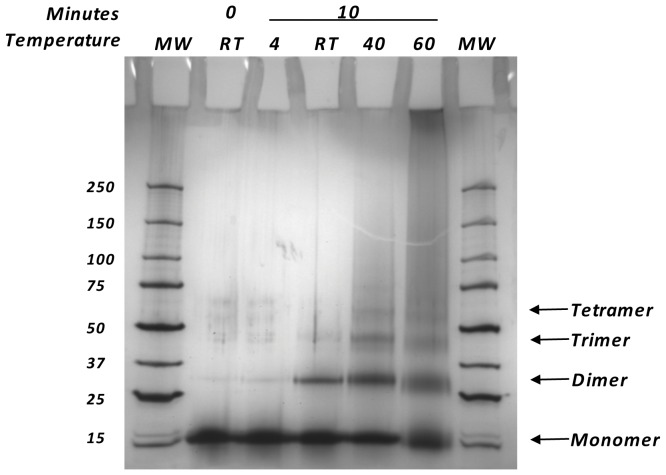
Crosslinking of RNase A. A 4–15% gradient SDS-PAGE showing crosslinking products of RNase A mixed with 10% neutral buffed formalin, for the times (minutes) and temperatures (degrees C) indicated; RT = Room Temperature. Outer Lanes: Precision Plus molecular weight (MW) markers, with molecular weights in kDa shown on the left, and the various oligomeric forms of RNase A indicated on the right.

Using an established model system used previously to study the reactivity of formalin in solution [43]–[45], we incubated RNase A in formalin at different temperatures and studied crosslinking by gel electrophoresis. While formalin chemistry is undoubtedly more complex in true tissues than in a solution of a purified protein, this model was chosen because it allows one to study a specific reaction, crosslinking, in isolation. Production of dimeric and oligomeric RNase A was moderate at room temperature, as expected, and the amount of crosslinking increased at elevated temperature (Figure 3). Conversely, the amount of crosslinking decreased dramatically at 4°C. These findings were interpreted as indicating that at lower temperatures, formalin may be able to penetrate into tissues in the relative absence of crosslinking reactions. A preincubation with low temperature formalin could, therefore, allow formalin to penetrate to the interior of a tissue specimen so that subsequently increasing the temperature would allow rapid fixation of both the periphery and center of a specimen.

To test this hypothesis, we fixed tonsil tissue in 4**°**C formalin for 2 hours prior to 40**°**C formalin for 10, 30 or 60 minutes (Figures 1 and 2, row B). For this experiment, as well as subsequent experiments, we used a 70% ethanol incubation after formalin fixation for samples that had shorter formalin exposure durations, so that all samples could then be loaded on the same downstream tissue processor for identical treatment. The duration of the 70% ethanol incubation after formalin fixation did not improve overall histologic appearance (data not shown). Compared to heated formalin only, we found that cold formalin pre-incubation improved histomorphology and immunoreactivity throughout a tissue specimen. Additional protocol optimization was performed on human tonsil using either no cold formalin preincubation or a 2 hour cold formalin preincubation, followed by 0.5, 1, 2, 4, or 6 hours in 35, 40, 45, or 50**°**C formalin. This subjective analysis indicated that the best results were obtained with 2 hours of 4**°**C formalin followed by 2 hours of 45**°**C formalin (see Figure S1). We termed this protocol “2+2”.

**Figure 4:**
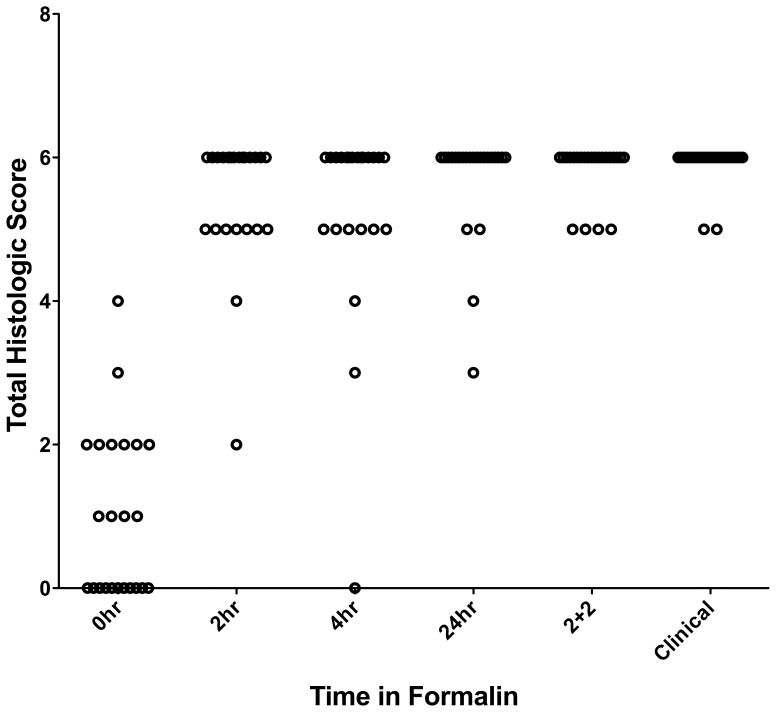
Histologic scores for each formalin fixation protocol, using criteria shown in Table 2. Time in formalin is listed for room temperature protocols, “2+2” is 2 hours in 4**°**C formalin followed by 2 hours in 45**°**C formalin, and “Clinical” refers to sections taken from blocks used for clinical diagnosis.

**Figure 5:**
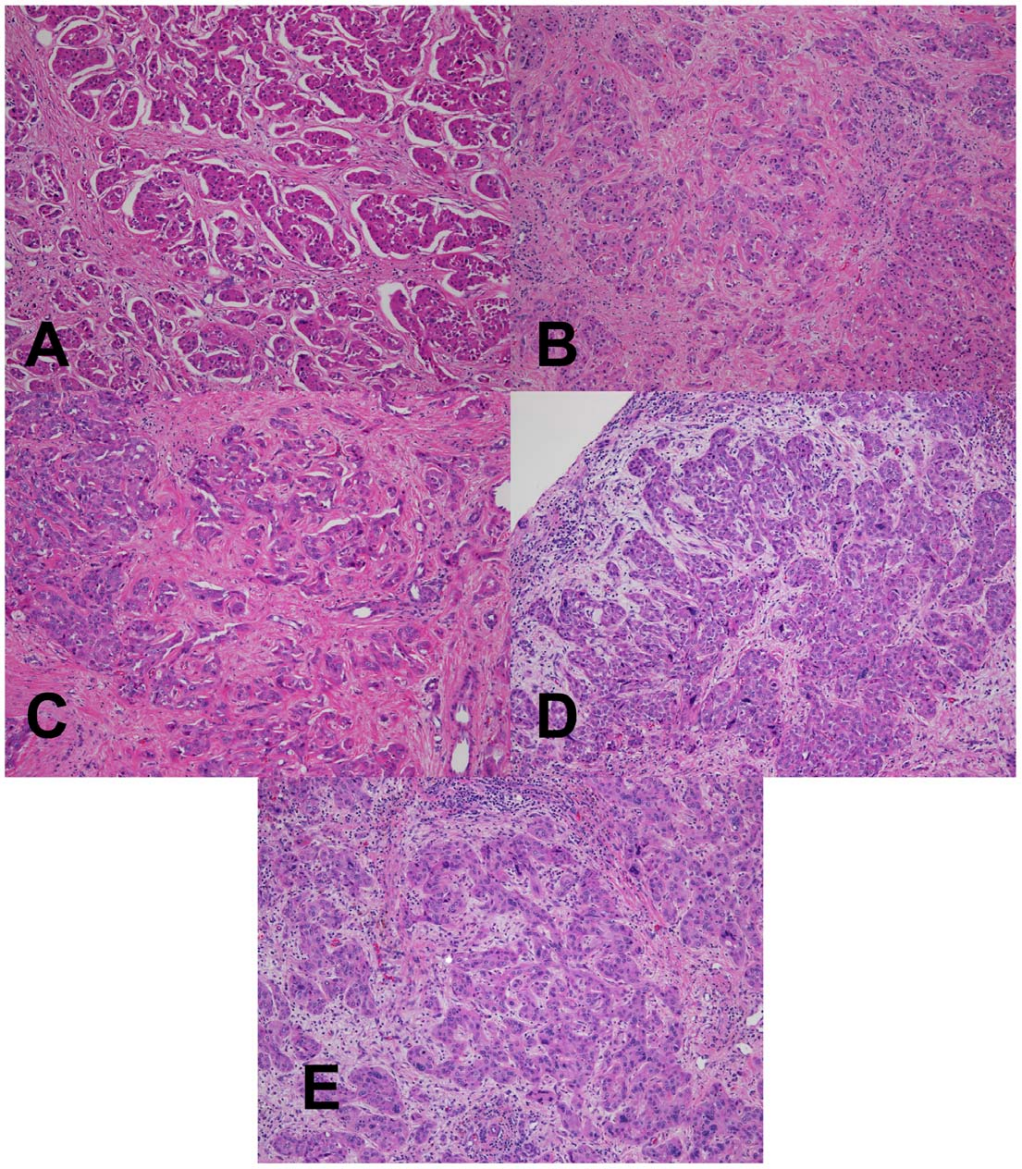
Low power magnification H&E analysis of fixed tumor specimen. (A–D) Hepatic cholangiocarcinoma fixed in room temperature formalin for 0, 2, 4, or 24 hr. (E) Hepatic cholangiocarcinoma fixed with 2+2 protocol. Images are 100× magnification.

**Figure 6:**
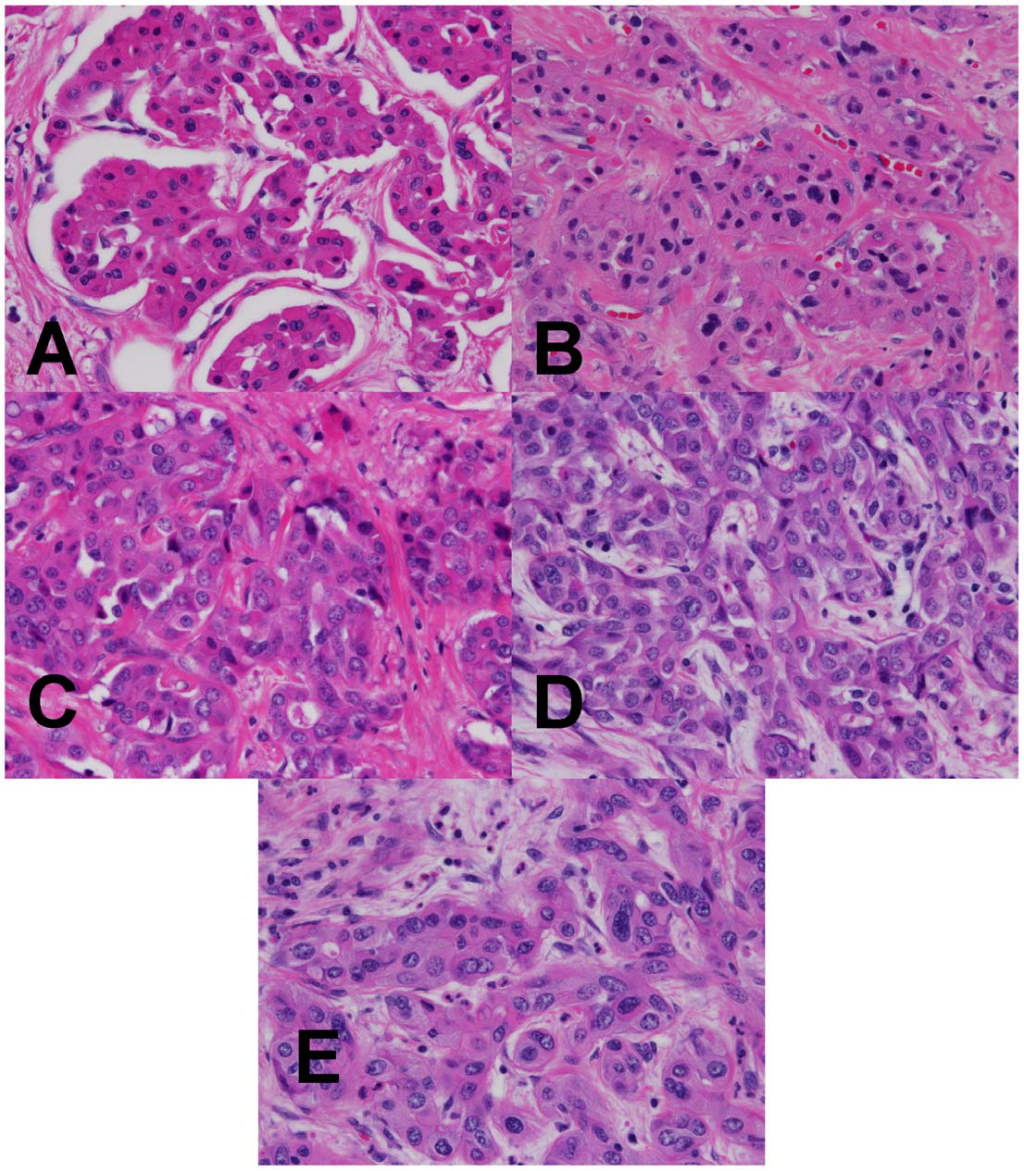
High power magnification H&E analysis of fixed tumor specimen. (A–D) Hepatic cholangiocarcinoma fixed in room temperature formalin for 0, 2, 4, or 24 hr. (E) Hepatic cholangiocarcinoma fixed with 2+2 protocol. Images are 400× magnification.

**Figure 7:**
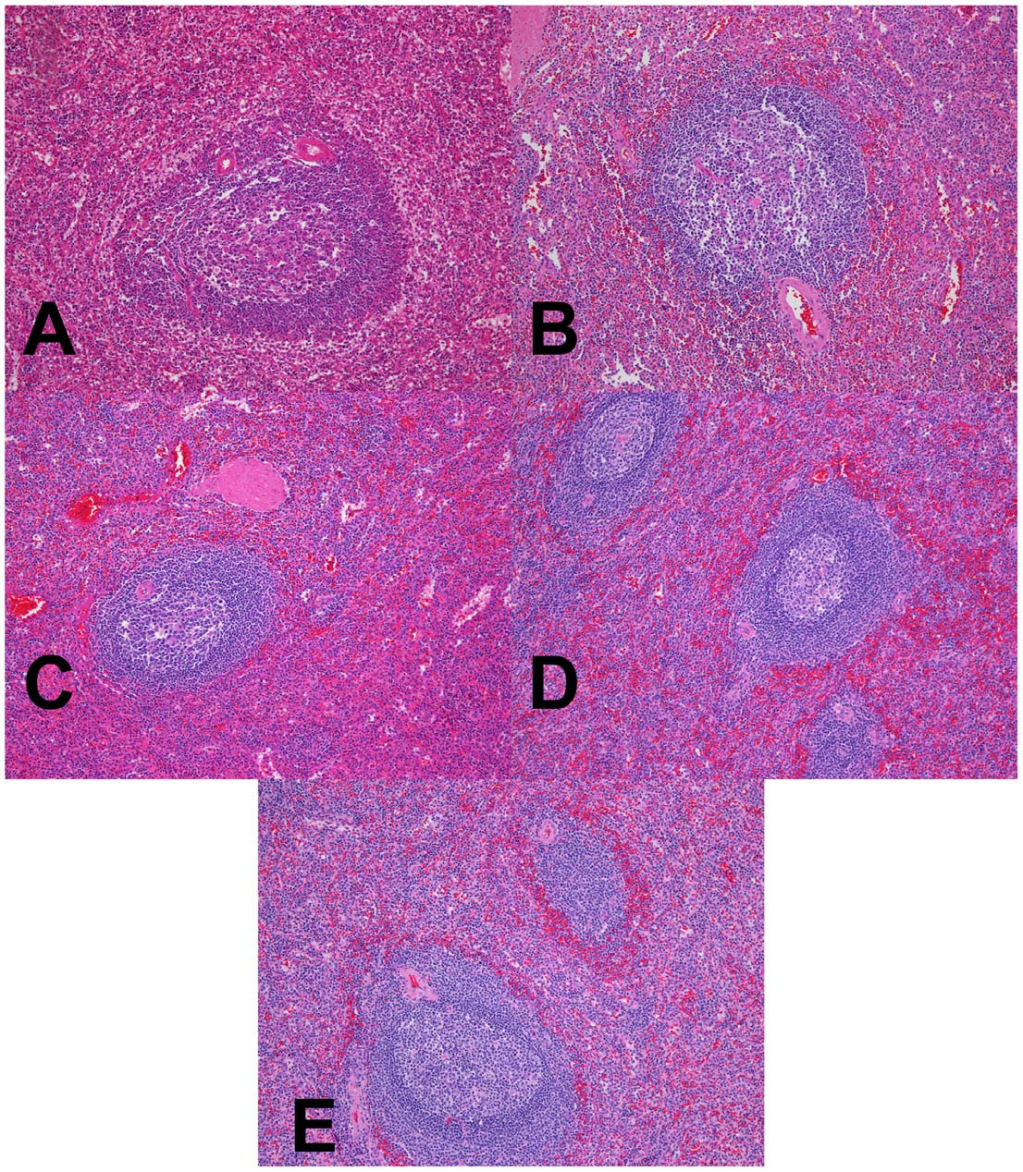
Low power magnification H&E analysis of fixed spleen. (A–D) Spleen fixed in room temperature formalin for 0, 2, 4, or 24 hr. (E) Spleen fixed with 2+2 protocol. Images are 100× magnification.

**Figure 8:**
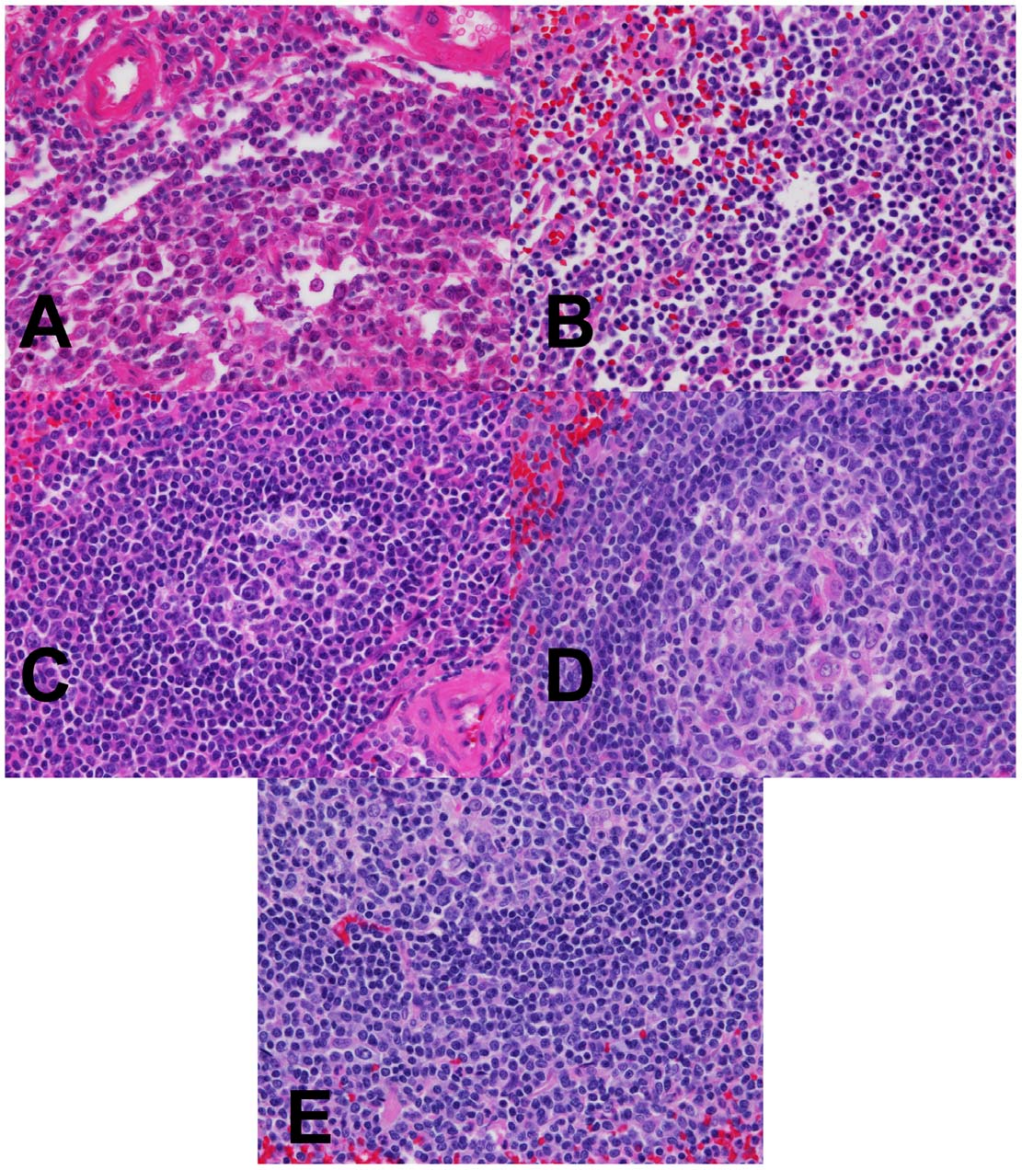
High power magnification H&E analysis of fixed spleen. (A–D) Spleen fixed in room temperature formalin for 0, 2, 4, or 24 hr. (E) Spleen fixed with 2+2 protocol. Images are 400× magnification.

To examine the performance of the 2+2 protocol on a broad range of the tissue types and downstream assays used in clinical pathology, we employed it in a clinical setting and compared it to a variety of fixation protocols. The tissues selected for this study were intended to represent a variety of tissue types encountered in general surgical pathology with different protein, lipid, and water contents (Table 1, n = 38 tissue samples tested with each condition). For this comparison, clinical tissue samples were fixed in room temperature formalin for 0, 2, 4, or 24 hours. The histomorphology and downstream assay performance from these protocols were compared with both the 2+2 protocol as well as tissue from the clinical block. Tissue histologic quality was scored by a combination of standard morphologic criteria and was found to depend on the time of fixation. Figure 4 shows the histologic scores from each condition, demonstrating that most samples with more than 2–4 hours of exposure to room temperature formalin had acceptable histology, with a (non-significant) trend towards improved histology with longer fixation times. Differences between all fixation protocols were significant by the Kruskal-Wallis with P<0.0001, with post-tests (Dunn’s Multiple Comparison Tests) indicating that 0 hour fixation was significantly inferior to all other conditions (P<0.05), but the differences between other conditions were not significant. No sample undergoing the 2+2 protocol had more than a single point deduction in the scoring scheme: samples of gastric antrum, colon and spleen each had a point deducted for overall morphology based on tissue “cracking” artifacts, and a single liver specimen had a point deducted for a mild cytosolic retraction artifact. Thus, the histomorphology observed over a wide variety of tissue types using the 2+2 protocol was indistinguishable from that obtained with overnight or 24 hour fixation, despite requiring 6–20 fewer hours. Representative examples of H&E sections from each fixation protocol are shown in Figures 5, 6, 7 and 8. Additional comparisons of 2+2 vs 24 hours room temperature fixation are shown for a wide variety of tissue types in Figures S2, S3 and S4.

**Figure 9:**
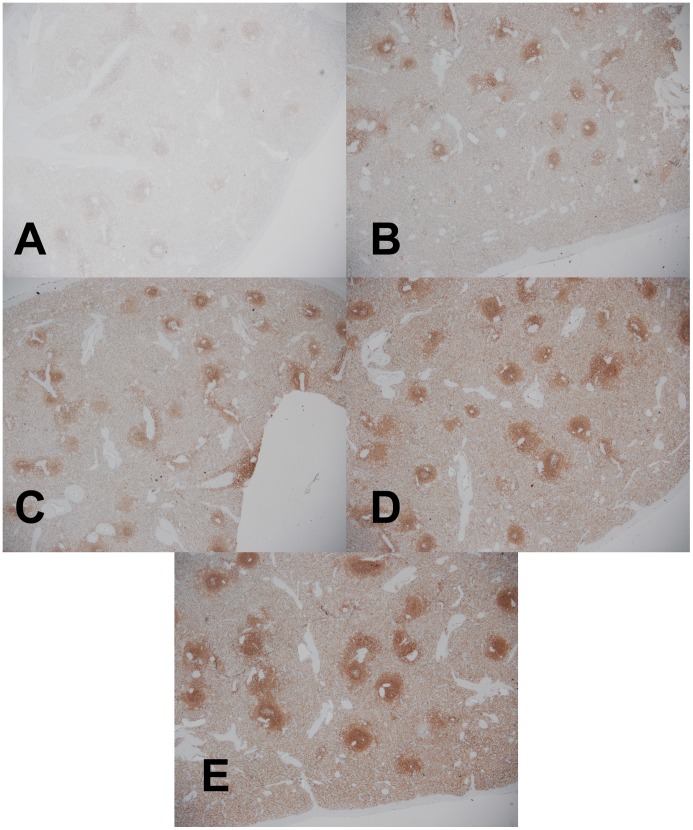
Low power magnification IHC analysis of fixed tissues. (A–D) Spleen fixed in room temperature formalin for 0, 2, 4, or 24 hr, respectively, and stained by IHC for bcl-2 expression. (E) Spleen fixed with 2+2 protocol and stained by IHC for bcl-2 expression. Images are 100× magnification.

**Figure 10:**
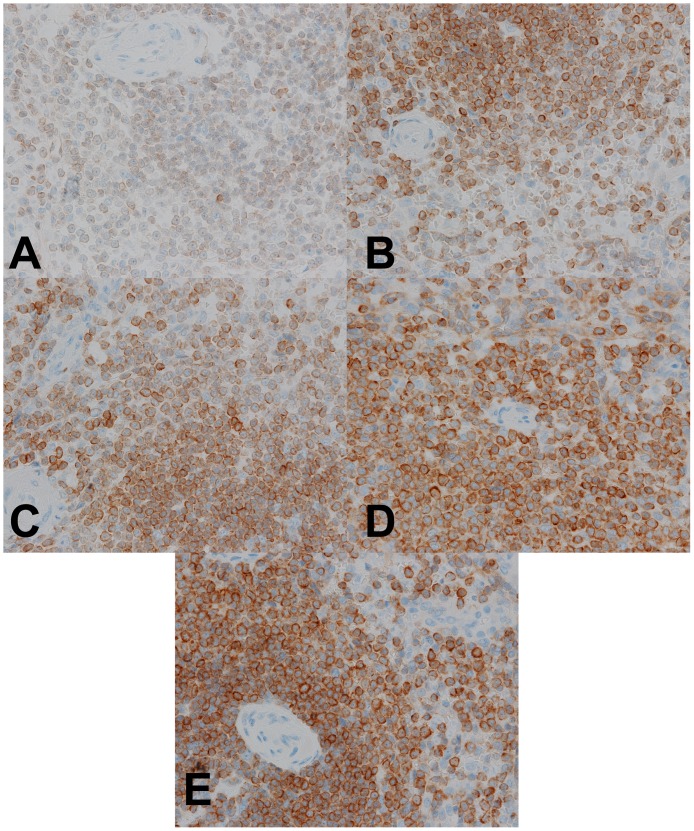
High power magnification IHC analysis of fixed tissues. (A–D) Spleen fixed in room temperature formalin for 0, 2, 4, or 24 hr, respectively, and stained by IHC for bcl-2 expression. (E) Spleen fixed with 2+2 protocol and stained by IHC for bcl-2 expression.

**Figure 11:**
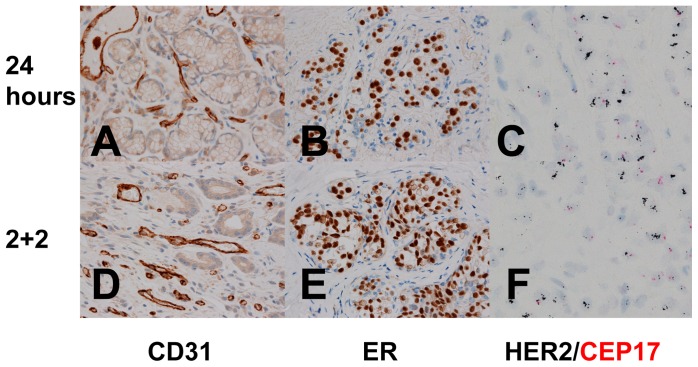
High Power IHC and ISH analysis of fixed tissues. (A, D) Gastric mucosa stained by IHC for CD31 expression. (B, E) Breast tissue with ductal carcinoma in situ stained by IHC for ER. (C, F) Breast tissue with ductal carcinoma in situ stained by DDISH for HER2 (black) and CEP17 (red). A, B and C are fixed in 24 hr room temperature formalin and D, E and F are fixed with the 2+2 protocol. All images are 400× magnification.

While our initial goal was to assess broadly the suitability of the 2+2 protocol for IHC analysis, few IHC assays are suitable or indeed interpretable in all tissue types collected in this study. Thus, only a few markers expressed in ubiquitous cell types (CD31 in vascular endothelium, vimentin in many cell types, *bcl-2* in lymphocytes) were assayed by IHC in all samples. A dual HER2/Chromosome 17 ISH was also performed in all samples to assess the performance of a nucleic acid-based test. In specific tissue types, additional tissue-specific IHC assays were run to better assess the performance of clinically relevant assays. For CD31, vimentin and bcl-2, qualitative IHC results were excellent, and indistinguishable, for all tissue samples whether fixed with the 2+2 protocol, 24 hours in room temperature formalin, or by the clinical laboratory’s standard protocol. Figures 9 and 10 demonstrate bcl-2 IHC on a spleen sample after all fixation conditions, and Figure 11 demonstrates CD31 and ER IHC and HER2 ISH compared between 2+2 and 24 hour room temperature fixation protocols. While semiquantitative analysis (i.e. H-score [46] or Allred Score [47]) is technically possible for these markers in the tissue types collected, the relevance of this approach is unclear because this sort of semiquantitative scoring was neither developed nor validated to accurately assess fixation quality. For example, while ER assessment on 2+2 and 24 hour room temperature tissue (Figures 11B and 11E) yielded identical Allred scores of 8, and while HER2 ISH performed on tissue treated with the same two fixation conditions (Figures 11C and 11F) both unambiguously demonstrated HER2 gene amplification, it is unclear how best to quantitate the difference, if any, between the two fixation conditions in the bcl-2 IHC assay (Figures 10D and 10E). We therefore chose to assess IHC staining qualitatively, and were unable to discern any decrement in staining in 2+2-treated tissue compared with 24 hour room temperature-treated tissue for these markers.

Representative IHC results from other common IHC markers (HER2 on breast DCIS and Vimentin on skin) after 2+2 and 24 hour room temperature fixation are shown in Figure S5. While testing of the tissue-specific markers HER2 and ER in a single sample of breast DCIS were likewise excellent and indistinguishable for the 2+2, 24-hour room temperature, or clinical fixation protocols, the fact that only a single sample of neoplastic breast tissue was available precluded statistical analysis of this finding.

**Figure 12:**
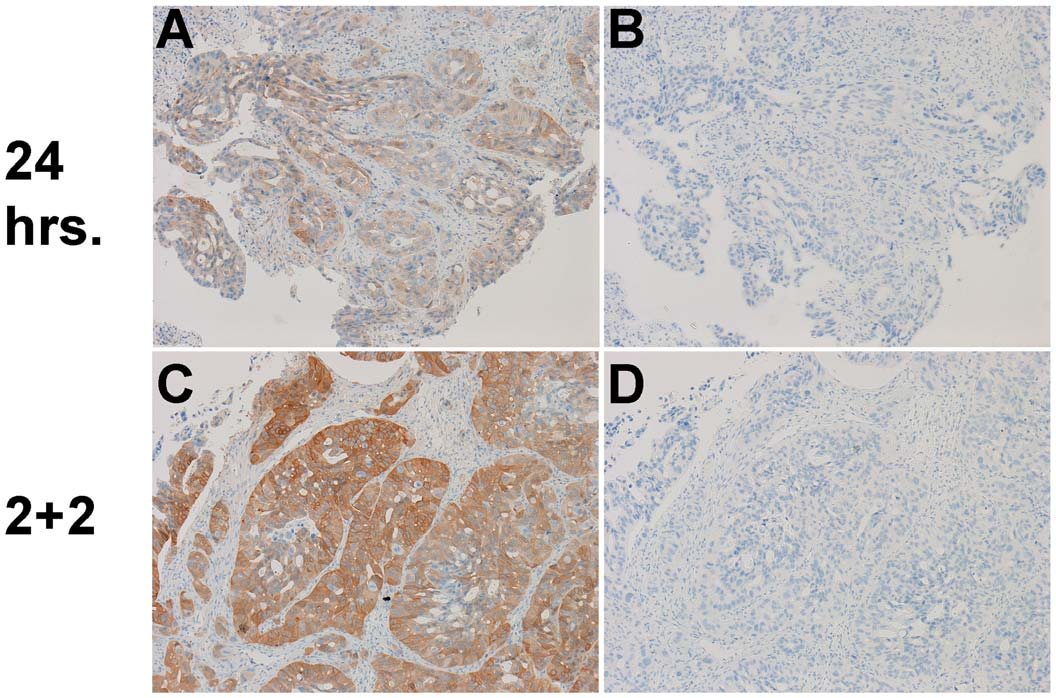
Phosphoprotein IHC. A, B); Calu-3 xenograft tissue fixed for 24 hrs in room temperature formalin and stained with pAKT antibody (CST #4060), without (A) or with (B) prior lambda phosphatase treatment.. C, D) Calu-3 xenograft tissue fixed with 2+2 protocol and stained with pAKT antibody, without (C) or with (D) prior lambda phosphatase treatment. All images are 400× magnification.

**Figure 13:**
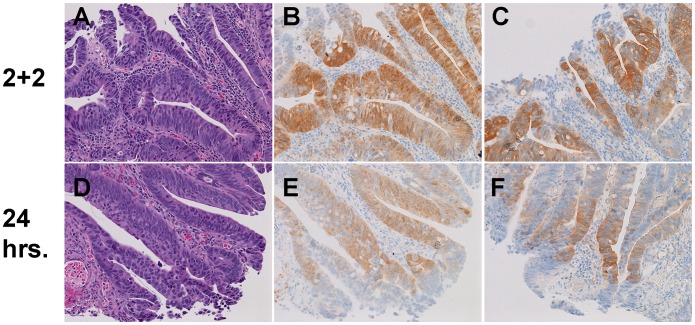
Phosphoprotein IHC. A–C) Human Colon Carcinoma fixed with 2+2 formalin protocol, stained with H&E (A), pAKT IHC (B), or pPRAS40 IHC (C). D-F) Human Colon Carcinoma fixed with 24 hours room temperature formalin, stained with H&E (D), pAKT IHC (E), or pPRAS40 IHC (F). All images are 400× magnification.

To test if the 2+2 protocol could preserve post-translational modifications, we next stained a variety of tissues with phosphoepitope-specific antibodies. Specific attention was focused on the phosphorylated form of the cancer biomarker AKT (pAKT), which had been challenging to detect in our past experience. We first stained a Calu-3 xenograft (known to overexpress the HER2 protein that activates AKT) with a pAKT-specific antibody (Figure 12). Much more pAKT staining was evident in the 2+2 samples compared to samples fixed for 24 hr at room temperature, and although staining is largely cytoplasmic, some membranous enhancement is evident in Figure 12C. Since phospho-markers can be relatively labile, we obtained human colonic adenocarcinoma samples from a commercial vendor, Indivumed, with low cold ischemia times (typically less than 15 minutes). Similar results were obtained when these human colonic adenocarcinomas were stained with pAKT (Figure 13, B and E), and for a second phosphoepitope, pPRAS40 (Figure 13, C and F). While areas of tissue near the luminal surface are shown in Figure 13 to highlight the changes seen in neoplastic epithelium, the enhancement in staining was seen to extend several millimeters deeper on lower power magnification (data not shown). Very thick samples with deeply invasive neoplastic epithelium were not available in the sample set obtained from Indivumed, so the ability of the 2+2 protocol to preserve phosphoepitopes in very thick sections of colon carcinoma tissue could not be investigated.

**Figure 14:**
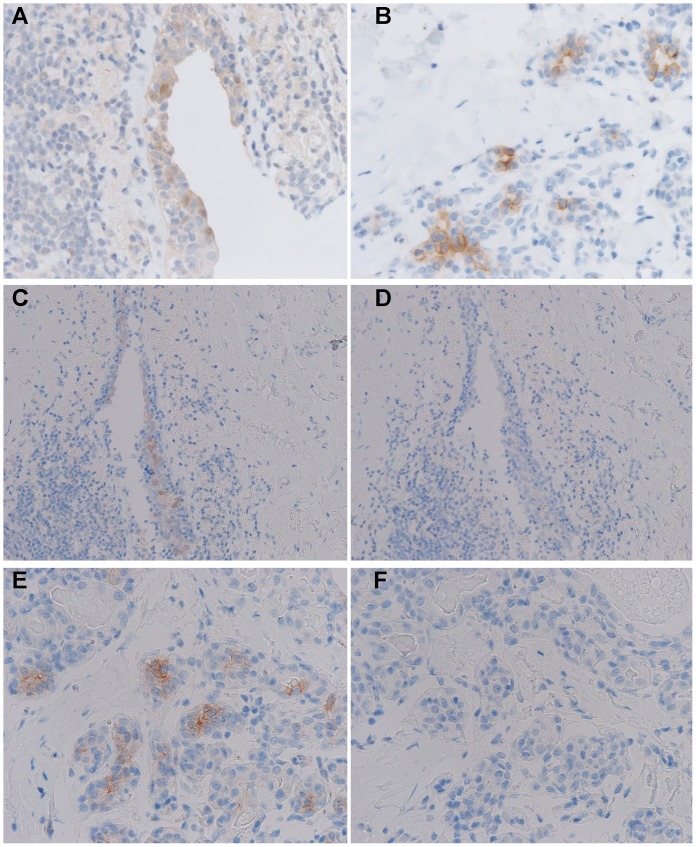
pAKT IHC on a human breast ductal carcinoma *in situ* (DCIS) case. A) 24-hour room temperature formalin fixation, 400× magnification, B) 2+2 protocol, 400× magnification, C,D) 24-hour room temperature formalin fixation, 100× magnification without (C) and with (D) lambda phosphatase treatment, E,F) 2+2 protocol, 400× magnification without (E) and with (F) lambda phosphatase treatment.

pAKT IHC staining on a clinical human breast DCIS sample, as well, was stronger and better localized to cell membranes with the 2+2 protocol compared to 24-hour room temperature fixation (Figure 14). The cells with membranous staining in Figure 14B and 14E were morphologically neoplastic yet adjacent to unstained lobular epithelial cells (i.e. within a so-called “cancerized” lobule [48]), indicating that the observed membranous staining, while weak, is indeed specific for a cancer cell-specific epitope and not a nonspecific artifact affecting all cells. In addition, in all cases of phosphoprotein IHC performed, staining was completely eliminated in adjacent sections treated with lambda phosphatase prior to antibody treatment, indicating that the antibodies were specifically recognizing the phosphorylated forms of proteins. Results from IHC employing a variety of other phosphoepitope-specific antibodies are shown in Figure S6, and demonstrate that the 2+2 protocol performs as well as 24-hour room temperature fixation for all markers tested.

**Figure 15:**
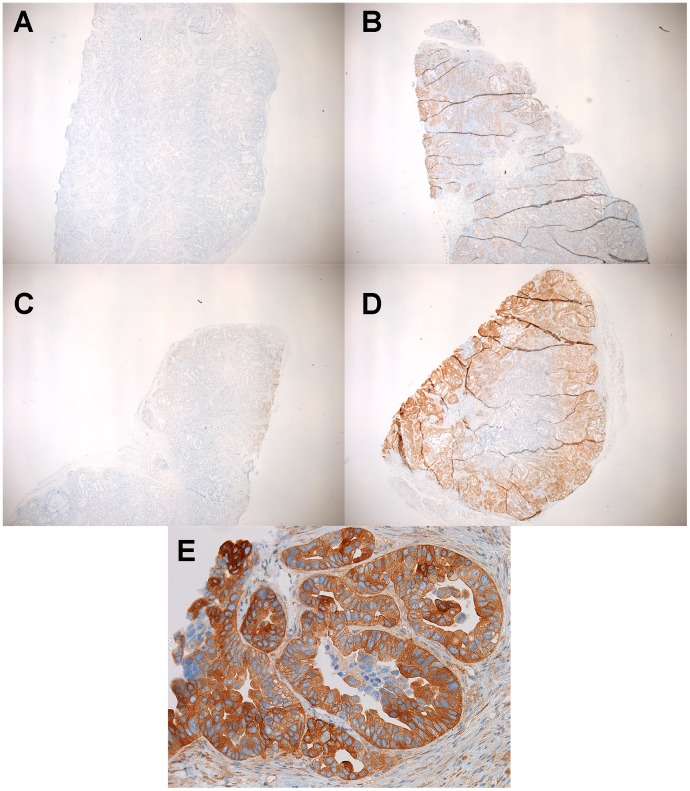
pAKT IHC in Calu-3 xenograft tumors with and without preincubation in cold formalin. A) No formalin fixation, B) 24-hour room temperature formalin, C) 2 hours 40**°**C formalin with no cold preincubation, D–E) 2+2 protocol. All images are at 40× magnification except for (E), which is at 400× magnification.

The necessity of the cold formalin preincubation step in the 2+2 protocol when applied to phosphoepitope IHC is demonstrated in Figure 15. In the Calu-3 xenograft, pAKT staining was strong and localized to tumor cytosol and cell membranes with the 2+2 protocol (Figure 15E), but it was almost completely obliterated (i.e. even less than seen with 24-hour room temperature fixation) when omitting the cold preincubation step.

## Discussion

We have developed a rapid formalin fixation protocol based on multiple temperature-controlled steps that substantially reduces fixation time from the standard ∼12–24 hours to 4 hours, while preserving histomorphologic and molecular information. While downstream tissue processing (dehydration, paraffin infiltration) steps can be lengthy and themselves introduce preanalytical morphologic and IHC artifacts, because we used identical tissue processing conditions in this study when making comparisons between fixation parameters, we believe that the effects that we describe here are specific to the fixation steps themselves, and not downstream processing steps.

A possible explanation for the utility of this protocol may be inferred from the results of formaldehyde crosslinking experiments with RNase A which showed that little, if any, crosslinking occurs at 4**°**C. This low activity at cold temperature could be simply due to a temperature-dependent kinetic slowing, or alternatively to the fact that formaldehyde exists in aqueous solutions in a temperature-dependent equilibrium with a hydrated form, methylene glycol, which has no crosslinking activity. Previous studies [4] have indicated that the ratio of formaldehyde to methylene glycol decreases greatly with temperature, i.e. approximately three-fold between 45**°**C and 20**°**C, and the ratio is expected to decrease even further at lower temperatures. Applying high temperature formalin directly to tissue, therefore, is expected to expose the periphery of the tissue to a high relative formaldehyde concentration, but the interior of the tissue will be rapidly warmed to potentially deleterious high temperatures before fixative is thoroughly distributed by diffusion. A cold preincubation with formalin, on the contrary, could allow time for diffusion of a high relative concentration of nonreactive methylene glycol to the interior of the tissue at a temperature where crosslinking is kinetically disfavored. A subsequent increase in temperature will drive the formaldehyde/methylene glycol equilibrium towards formaldehyde and increase the kinetics of crosslinking, thus quickly generating active formaldehyde throughout the tissue as it warms. An additional benefit of cold preincubation may be the expected relative reductions in the activities of degrading enzymes like phosphatases at lower temperatures.

The benefits of a two-step fixation process in which nonreactive formaldehyde is delivered to tissues prior to a subsequent activation step have been demonstrated previously [49], indicating that our proposed mechanism is plausible. In this previous report, formaldehyde was applied first at a low pH, at which it is inactive, followed by a shift to a high pH where formaldehyde is very active. While the pH-shifting strategy was effective at preserving the morphologic distribution of a single soluble protein, its generalizability to routine histology and IHC was not demonstrated, and it is unclear how phosphoepitopes would be preserved in the protocol.

Beyond increasing the overall speed of fixation, one of our goals in this effort was to devise a fixation strategy that preserved the medical utility of formalin-fixed, paraffin-embedded (FFPE) tissues for a broad variety of assays. Of course, it would be impossible to assess the performance of every possible IHC or molecular tissue assay on every type of tissue, so we chose to focus on a few relevant assays, and specifically IHC assays for phosphoproteins. That few, if any, IHC assays for phosphoproteins are performed in clinical pathology laboratories is probably due in large part to preanalytical variables that make such assays unreliable. No fixation protocol is likely to improve matters if other preanalytical variables (warm and cold ischemia times, tissue cut too thickly for formalin penetration, etc…) are poorly controlled, but several assays such as phosphoepitope IHC are known to be problematic even when pre-fixation conditions are rigorously standardized. The Calu-3 xenograft and human colon carcinoma samples studied here were collected in a research environment and hence amenable to strict control of ischemia time, but our clinical breast DCIS sample was collected when the specimen underwent routine gross dissection more than 30 minutes after excision with no record of warm ischemic time, indicating that the 2+2 protocol might improve downstream assay performance even in a clinical setting. Proving enhanced performance of phosphoprotein IHC assays with the 2+2 protocol will require a specific clinical trial where ischemic times are well defined, but the utility of such a finding may be less relevant to clinical pathology than to research applications because ischemic times are often difficult to control or even measure in clinical settings. Studying phosphoprotein IHC assays with 2+2 fixation will also require a validated scoring system, which to date does not exist for all such assays.

To date, the 2+2 protocol has been investigated in conjunction with 14 different phospho-specific IHC assays performed on either Calu-3 xenografts, breast DCIS, or colon carcinoma samples (examples shown in Figure S6, with phosphatase-treated control samples to demonstrate specificity). The results of these studies indicate that some phosphoepitopes (pAKT and pPRAS40) are extremely labile and appear to benefit greatly from the 2+2 protocol, whereas other phosphoepitopes appear to be stable and can be detected in standard fixation protocols.

While we have not exhaustively demonstrated the compatibility of the 2+2 protocol with every tissue type and potential tissue-based assay, we have yet to encounter any tissue or specific downstream assay for which the 2+2 protocol negatively impacts performance. To the contrary, we have demonstrated that the 2+2 protocol is indistinguishable from lengthy standard room-temperature formalin fixation for a broad range of tissues and applications, including standard histomorphologic study, standard and phosphoepitope IHC and at least one ISH assay. Numerous clinical assays are currently performed on formalin-fixed tissues, however, including IHC for prognostic and predictive markers, gene sequencing and amplification-based nucleic acid assays. The applicability of the 2+2 protocol for these assays thus requires further investigation and clinical validation, especially where quantitative or semi-quantitative assays of tissue protein expression by IHC influence clinical decisions. A recent study [3] indicating the superiority of cold formalin fixation for nucleic acid preservation suggests that the 2+2 protocol may indeed be worth investigating in these areas.

## Supporting Information

Figure S1 **H&E and bcl-2 IHC analysis of human tonsil tissue.** H&E and bcl-2 IHC analysis of human tonsil tissue fixed for varying times at elevated temperatures with and without a preincubation in 4**°**C formalin. Histologic quality is indicated by a color code corresponding to a pathologist’s subjective analysis: red = “unacceptable”, yellow = “acceptable for diagnosis but with recognizable defects in morphology”, and green = “acceptable”. To ensure reproducibility in subjective scoring, control samples were fixed in room temperature formalin for 0, 2, 4, 8 and 24 hours and used for comparisons. The H&E chart is a summary of analysis of 10 different samples, and the bcl-2 IHC chart is a summary of data from 2 samples.
(TIF)

Figure S2 **H&E analysis of 24 hour fixation vs. 2+2 specimens.** Cecum, cervical carcinoma, and metastatic colon cancer (sampled from liver) are shown for each condition.
(TIF)

Figure S3 **H&E analysis of 24 hour fixation vs. 2+2 specimens.** Endometrium, fallopian tube, and a uterine leiomyoma are shown for each condition.
(TIF)

Figure S4 **H&E analysis of 24 hour fixation vs. 2+2 specimens.** Liver, skeletal muscle, and skin are shown for each condition.
(TIF)

Figure S5 **IHC analysis of 24 hour fixation vs. 2+2 specimens.** HER2 IHC on a HER2-overexpressing breast DCIS sample and vimentin IHC on skin are shown for each condition.
(TIF)

Figure S6 **Phosphoprotein IHC.** Phosphoprotein IHC performed on colonic adenocarcinoma samples fixed with 2+2 protocol (leftmost columns) or 24 hours in room temperature formalin (rightmost columns). Rows are labeled with the phosphoprotein antibody used, and for each fixation protocol, sections treated with or without phosphatase are shown to indicate specificity.
(TIF)
