# Supplementary figures and images for: Rapid Two-Temperature Formalin Fixation
Source: PLoS One. 2013 Jan 18;8(1):e54138. doi: 10.1371/journal.pone.0054138 (PMC3548901; doi:10.1371/journal.pone.0054138)

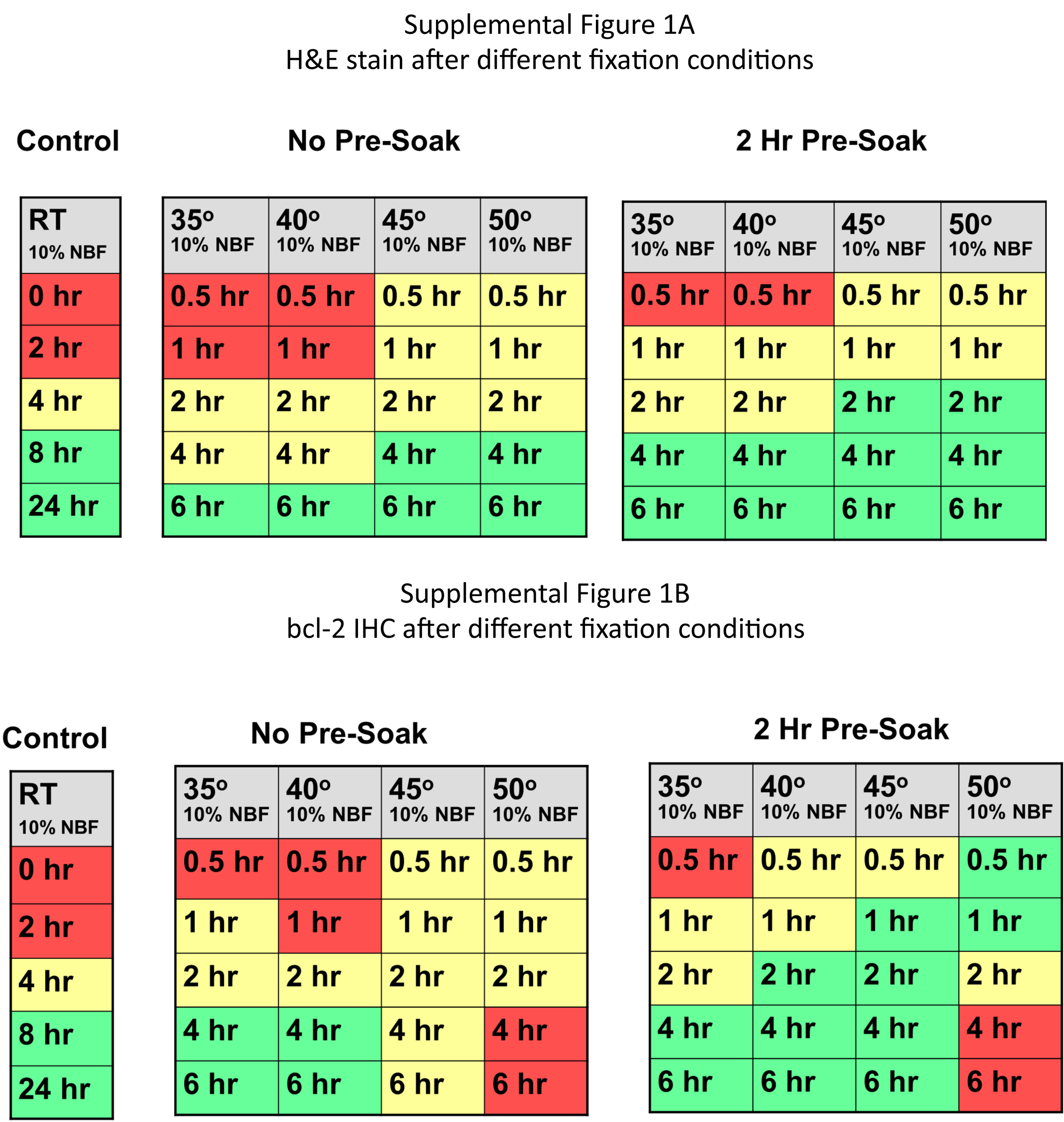

Supplement: Figure S1 — H&E and bcl-2 IHC analysis of human tonsil tissue. H&E and bcl-2 IHC analysis of human tonsil tissue fixed for varying times at elevated temperatures with and without a preincubation in 4°C formalin. Histologic quality is indicated by a color code corresponding to a pathologist’s subjective analysis: red = “unacceptable”, yellow = “acceptable for diagnosis but with recognizable defects in morphology”, and green = “acceptable”. To ensure reproducibility in subjective scoring, control samples were fixed in room temperature formalin for 0, 2, 4, 8 and 24 hours and used for comparisons. The H&E chart is a summary of analysis of 10 different samples, and the bcl-2 IHC chart is a summary of data from 2 samples. (TIF) [file pone.0054138.s001.tif]

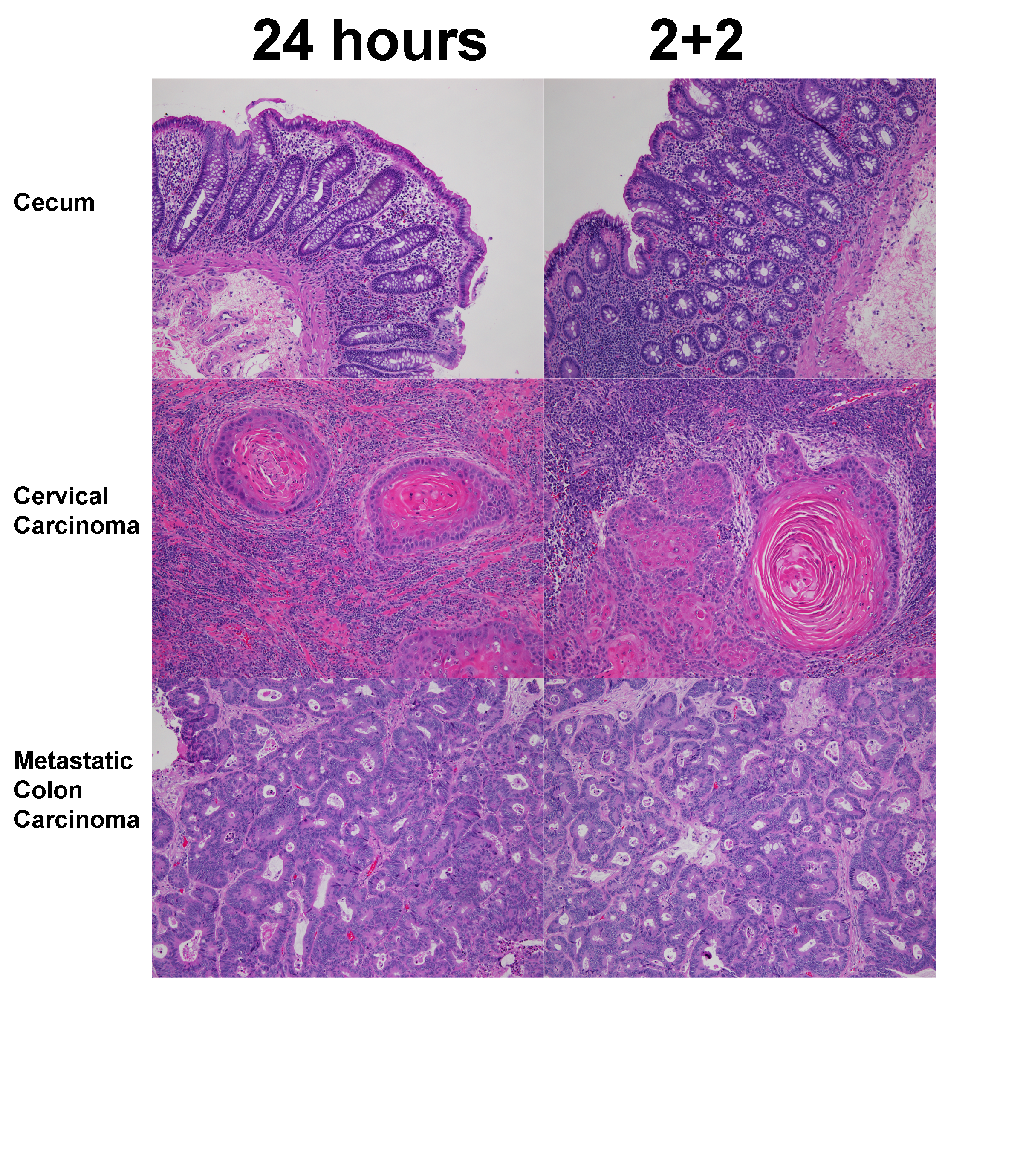

Supplement: Figure S2 — H&E analysis of 24 hour fixation vs. 2+2 specimens. Cecum, cervical carcinoma, and metastatic colon cancer (sampled from liver) are shown for each condition. (TIF) [file pone.0054138.s002.tif]

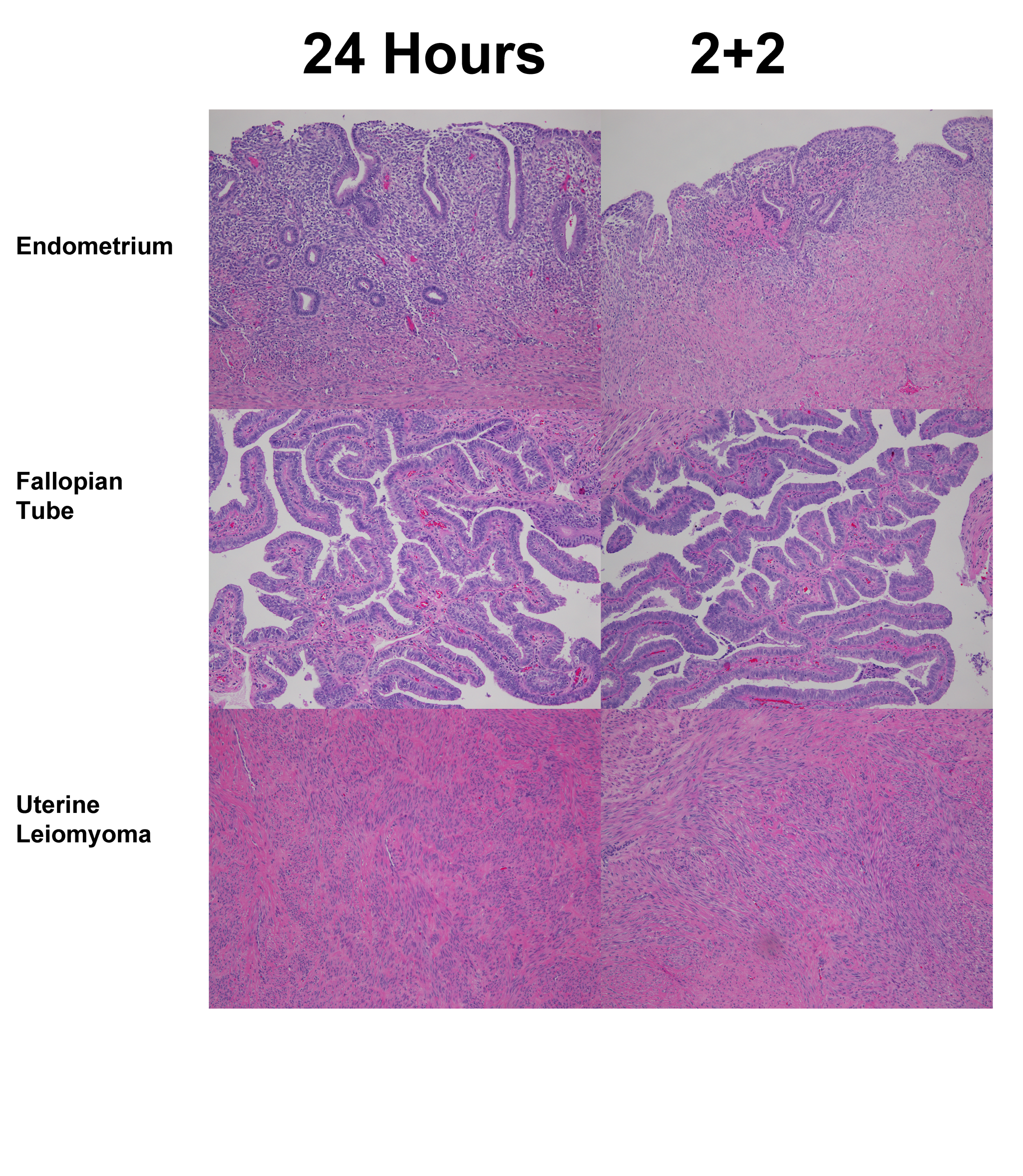

Supplement: Figure S3 — H&E analysis of 24 hour fixation vs. 2+2 specimens. Endometrium, fallopian tube, and a uterine leiomyoma are shown for each condition. (TIF) [file pone.0054138.s003.tif]

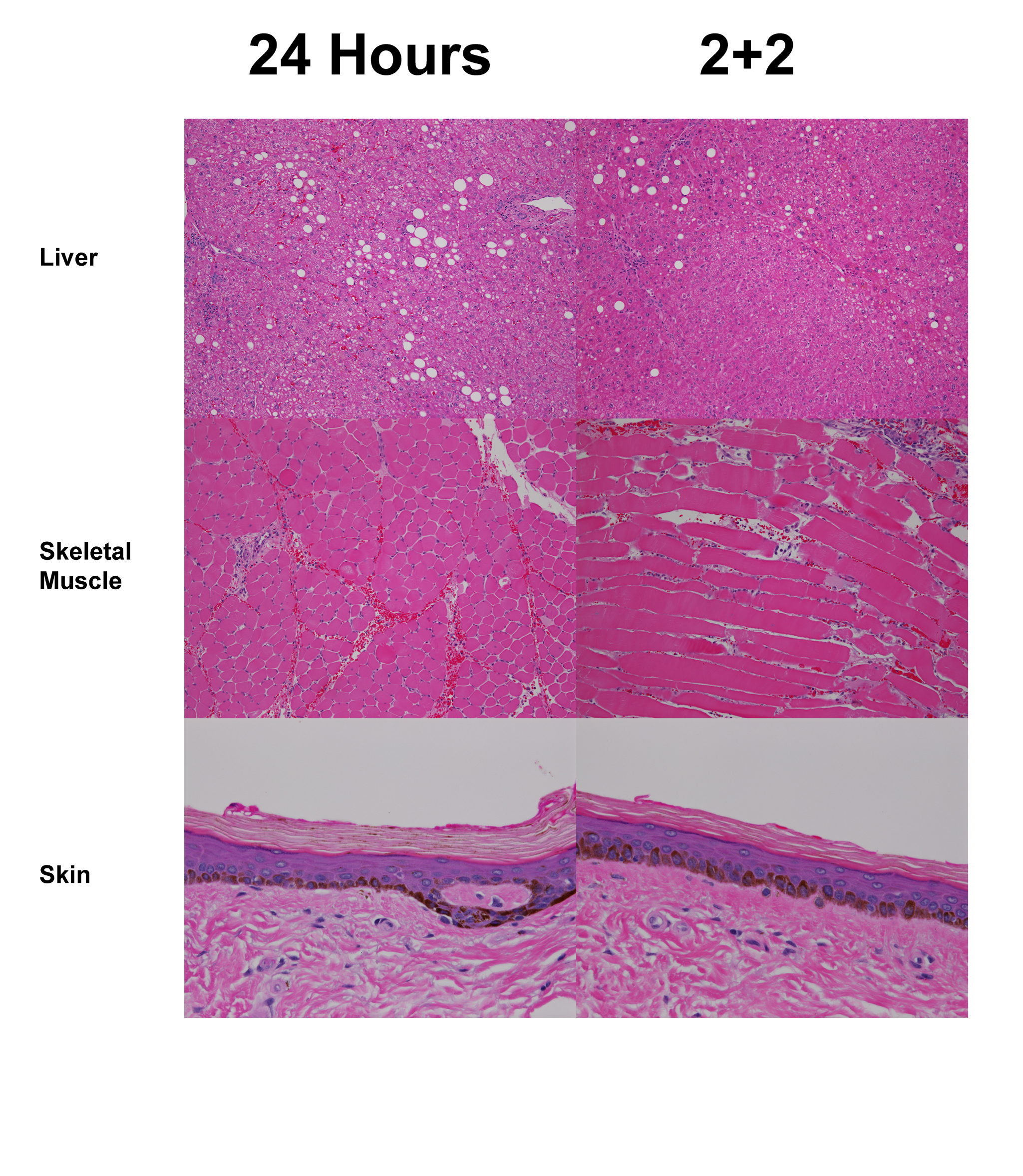

Supplement: Figure S4 — H&E analysis of 24 hour fixation vs. 2+2 specimens. Liver, skeletal muscle, and skin are shown for each condition. (TIF) [file pone.0054138.s004.tif]

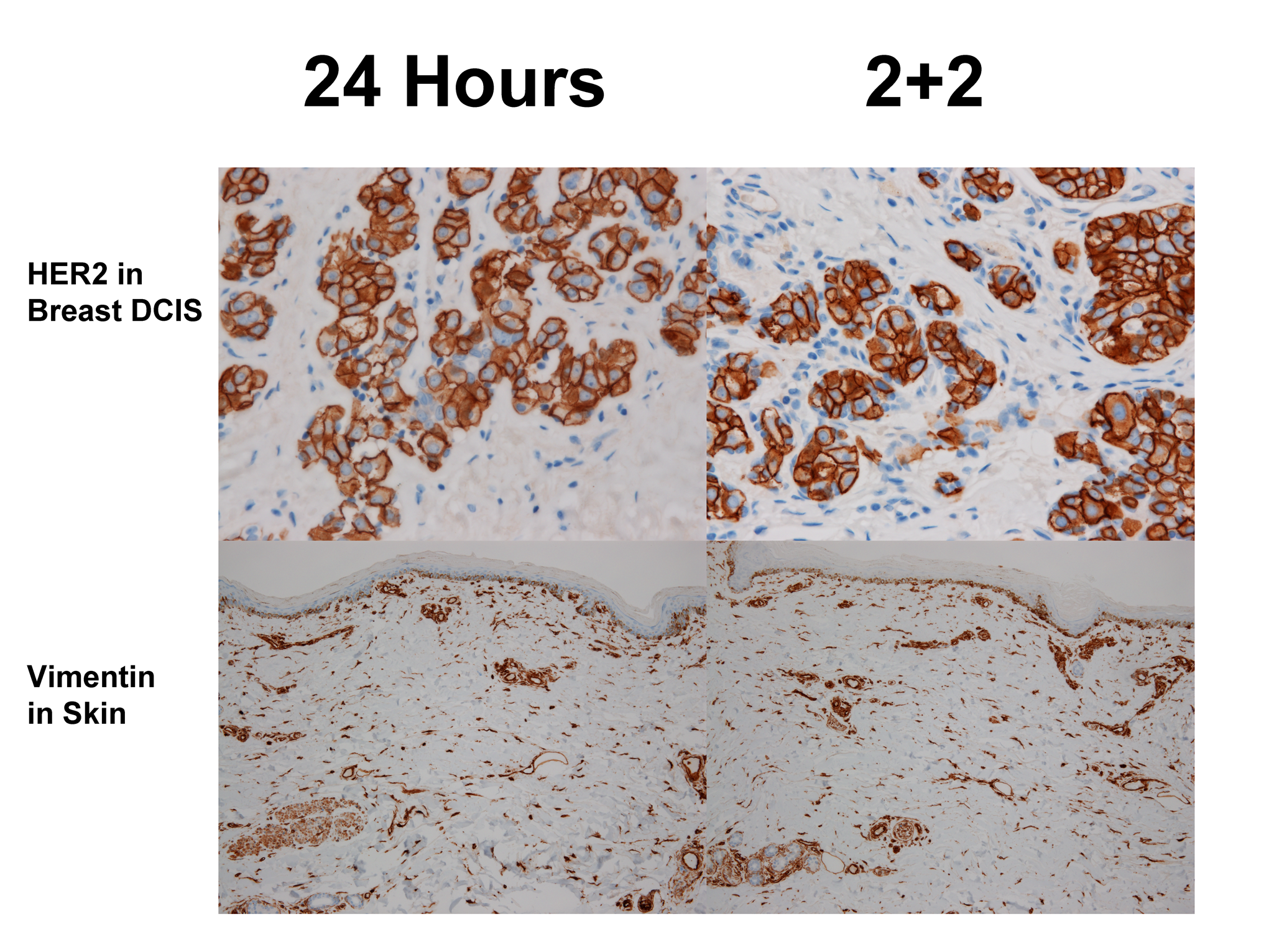

Supplement: Figure S5 — IHC analysis of 24 hour fixation vs. 2+2 specimens. HER2 IHC on a HER2-overexpressing breast DCIS sample and vimentin IHC on skin are shown for each condition. (TIF) [file pone.0054138.s005.tif]

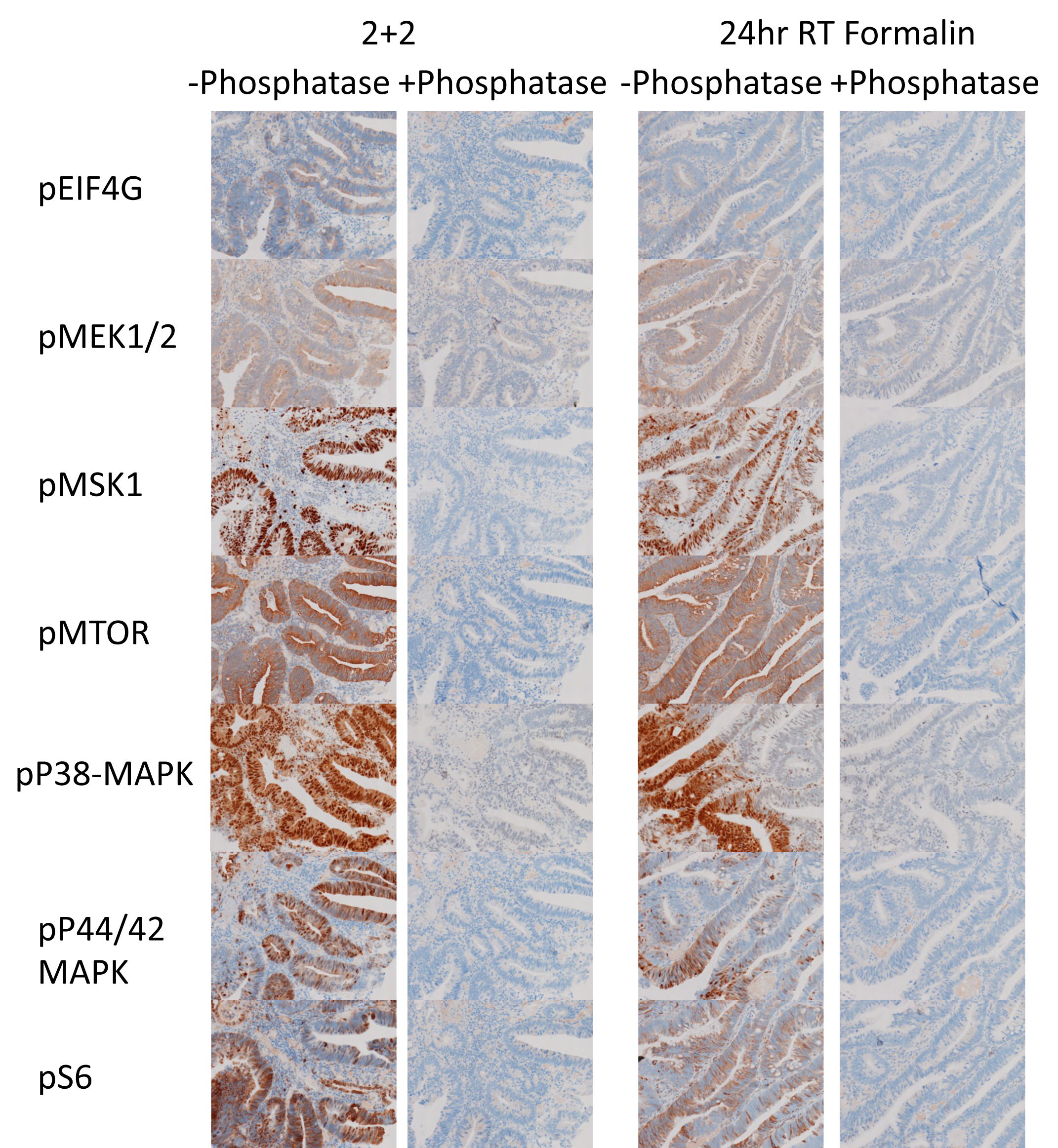

Supplement: Figure S6 — Phosphoprotein IHC. Phosphoprotein IHC performed on colonic adenocarcinoma samples fixed with 2+2 protocol (leftmost columns) or 24 hours in room temperature formalin (rightmost columns). Rows are labeled with the phosphoprotein antibody used, and for each fixation protocol, sections treated with or without phosphatase are shown to indicate specificity. (TIF) [file pone.0054138.s006.tif]
